# Shoulder dislocations in professional male football (soccer) do not negatively affect long‐term quantitative and qualitative performance parameters: A retrospective analysis of 30 in‐match injuries of the German Bundesliga

**DOI:** 10.1002/jeo2.70589

**Published:** 2025-12-07

**Authors:** Blanca Julie Degener, Christoph Theil, Georg Gosheger, Patrick May, Jörg Seidel, Tim Schachtrup, Theodoros Zafeiris, Kristian Nikolaus Schneider

**Affiliations:** ^1^ Department of Orthopaedics and Tumor Orthopaedics University Hospital of Münster Münster Germany; ^2^ Goal Impact GmbH Hamburg Germany

**Keywords:** Bundesliga, dislocation, football, performance, shoulder, soccer

## Abstract

**Purpose:**

Shoulder dislocations in professional football are increasing and regularly associated with substantial layoff times. However, research on players' postinjury performance remains limited. Therefore, the purpose of this study was to evaluate whether first‐time shoulder dislocations negatively affect performance after return to play (RTP) and to determine whether the type of treatment (nonoperative vs. operative) influences post‐injury performance outcomes.

**Methods:**

Retrospective, media‐based analysis of all first‐time in‐match shoulder dislocations in the top two German football leagues (first and second Bundesliga) from the 2012/2013 to 2021/2022 season (*n* = 30). Quantitative and qualitative performance data, as well as player‐, match‐ and injury‐related data were obtained from official databases. The Goalimpact (Goalimpact GmbH), a numerical value representing an individual player's impact on team success, served as the qualitative parameter. Data were analysed up to 2 years pre‐injury and 2 years post‐RTP. Statistical significance was set at *p* < 0.05.

**Results:**

A total of 30 shoulder dislocations in 30 players with a median age of 25 years (Interquartile range [IQR]: 23–26) were available for analysis. Median layoff time was 65 days (IQR: 23–115), 22 days (IQR: 10–34) for those treated conservatively and 112 days (IQR: 92–133; *p* < 0.001) for players undergoing surgery. Quantitative performance parameters showed a temporary decrease, with fewer matches (*p* = 0.017) and minutes played (*p* = 0.004) in the 10 matches following RTP compared to the 10 matches pre‐injury. Goalimpact values were initially comparable to pre‐injury levels but improved 1 year (131 [IQR: 115–150] vs. 141 [IQR: 122–160]; *p* = 0.001) and 2 years (127 [IQR: 107–153] vs. 139 [IQR: 124–161]; *p* < 0.001) after RTP compared to pre‐injury. No significant differences in performance were found regarding type of injury or type of treatment, nor were performance outcomes statistically significantly influenced by player's age, league and position played.

**Conclusion:**

Shoulder dislocations in professional football led to a temporary short‐term decrease in quantitative performance parameters, while qualitative performance parameters remained initially similar. Type of injury as well as type of treatment, player's age, league and position played did not significantly influence performance outcomes.

**Level of Evidence:**

Level III.

**Abbreviations**
ACL: anterior cruciate ligament
IQR: interquartile range
MSK: musculoskeletal
NBA: National Basketball Association
NHL: National Hockey League
PER: player efficiency rate
RTC: return to competition
RTP: return to play

## INTRODUCTION

Injuries in professional football are the major cause of absences and negatively impact team performance [12, 17]. The incidence of shoulder dislocations within professional football is increasing and currently amounts to 9.1 per 1000 matches played [21]. While this rate is relatively low compared to lower‐extremity injuries, shoulder dislocations remain a significant concern: A recent study found that shoulder dislocations within the German Bundesliga lead to a median layoff time of 79 days and nearly a quarter of players who experienced a first‐time shoulder dislocation suffered a recurrent instability event [1, 21].

Contrary to other injuries in professional football, the ideal treatment following a shoulder dislocation remains controversial [21]. Besides medical factors such as concomitant injuries, the likelihood of a re‐dislocation event or the time to return to competition (RTC), nonmedical factors like the player's post‐injury quantitative and qualitative performance are of key interest in the decision‐making process. However, the term ‘performance’ in professional football is broadly defined and various parameters are available for assessment. Lately, algorithms like the Goalimpact have been developed to measure not only the individual performance but also each player's contribution to overall team performance. Given that prospective assessment using clinical scores is not feasible in professional football due to the confidential nature of medical records and the retrospective design of this study, the Goalimpact algorithm provides a valuable alternative by objectively quantifying on‐field performance through systematic analysis of match data across multiple performance parameters.

There is a paucity of research on performance outcomes following shoulder dislocations in professional football, with only limited data available for other professional sports. Although prior studies have examined performance following shoulder dislocations in upper‐extremity focused sports, there remains a gap in the literature regarding this topic in lower‐extremity sports such as football. In the National Basketball League (NBA), Li et al. found that nonoperative treatment following a shoulder instability event leads to a reduced player efficiency rate, fewer games played and lower win shares [14]. Similarly, in the National Hockey League (NHL), shoulder instability events resulted in high RTC rates and acceptable performance outcomes [22]. However, while it appears that shoulder dislocations lead to reduced player performance in upper extremity‐type sports, it remains unclear whether these findings translate to professional football, where the biomechanical demands and performance metrics differ substantially from upper‐extremity sports.

Given this research gap, this study aimed to evaluate (1) quantitative as well as (2) qualitative performance parameters and (3) to identify relevant player‐, match‐ and injury‐related factors that affect these parameters following a shoulder dislocation in professional football. We hypothesised that first‐time shoulder dislocations in professional football do not negatively affect the individual quantitative and qualitative performance parameters.

## MATERIALS AND METHODS

The study was approved by the local ethics committee (reference number: 2023‐687‐f‐S) and was conducted in accordance with the Declaration of Helsinki.

This study is a retrospective analysis of all in‐match shoulder dislocations occurring in the top two German football leagues (first and second Bundesliga) from the 2012/2013 season to the 2021/2022 season. Players with recurrent shoulder dislocations (*n* = 7) were excluded, leaving a total of 30 first‐time in‐match shoulder dislocations among 30 players for further analysis (Table 1). Shoulder dislocations were identified performing a media analysis previously proposed and validated by Krutsch et al. [13]. Information regarding concomitant injuries in these players is not publicly available and was thus not available for analysis. For primary outcomes, quantitative performance parameters were obtained using the official Bundesliga database (www.bundesliga.de) as well as a publicly available football statistics database (www.transfermarkt.de) previously used for research in professional football [10, 20, 21]. For secondary outcomes, qualitative performance data were obtained from Goalimpact, a global player rating agency (Goalimpact GmbH). Quantitative parameters were collected for 2 seasons, 1 season and 10 matches prior to the injury and following RTC, respectively. Qualitative parameters were obtained for 2 years, 1 year, 2 months and 1 month before injury and after RTC, respectively.

**Table 1:** Player‐, match‐ and injury‐related variables.

Variable	*n* (%)
Player‐related	
Age	
<25	14 (47)
>25	16 (53)
Position played	
Defense	14 (47)
Midfield	9 (30)
Offense	8 (23)
League played	
1. Bundesliga	13 (43)
2. Bundesliga	17 (47)
Match‐related	
Season	
First half of the season	13 (43)
Second half of the season	17 (57)
Injury‐related	
Mechanism of injury	
Type 1	11 (38)
Type 2	18 (62)
Type of treatment	
Nonoperative	18 (60)
Operative	12 (40)

### Quantitative performance parameters

Quantitative performance parameters included matches and minutes played as well as goals scored and assists made.

### Qualitative performance parameters

Qualitative performance parameters included are the Goalimpact and the Peak Goalimpact, an age‐adjusted measure reflecting the potential maximum of an individual player.

The Goalimpact index measures each player's contribution to their team's goal difference and adjusts for potential confounders (e.g., red cards and home‐field advantage). Unlike traditional, single‐dimension performance indicators, the Goalimpact provides an aggregated assessment of a player's influence on team success. The metric is given as a monthly, numerical value, with higher values indicating a better player rating. Given that the Goalimpact is published monthly, qualitative performance comparisons were made for 2 years, 1 year, 2 months and 1 month prior to the dislocation event and after RTC.

### Player‐, match‐ and injury‐related information

Player‐related data included players' age, position and league played.

Match‐related details encompassed the first half versus the second half of the season of injury.

Regarding injury‐related information, the injury mechanisms were classified according to Schneider et al.: Type 1 shoulder dislocations for direct contact injuries, type 2 for dislocations caused by catching a fall [20]. In addition, the subsequent treatment (nonoperative vs. operative) and respective layoff times were obtained.

### Statistics

For statistical analysis, data normality was determined using the Kolmogorov–Smirnov test. Data not following a normal distribution are described as median along with the 25%–75% interquartile range (IQR), whereas normally distributed data are presented as mean with their ranges. Given the nonparametric data distribution, nonparametric tests were used to compare groups: the Mann–Whitney *U*‐test for comparing two independent samples or the Kruskal–Wallis test for comparing more than two independent samples. For paired observations, the Wilcoxon signed‐rank test was employed to determine differences before and after injury events. A *p*‐value of less than 0.05 was considered statistically significant.

## RESULTS

### Quantitative parameters

Following RTC, players competed in significantly fewer matches over 10 match days compared with the 10 match days leading up to shoulder dislocation (8 [IQR: 6–9] vs. 9 [IQR: 8–10]; *p* = 0.017). Likewise, the total number of minutes played was significantly reduced after RTC (571 [IQR: 301–702] vs. 698 [IQR: 459–842]; *p* = 0.004) (Table 2). The mean follow‐up time was approximately 672 days.

**Table 2:** Quantitative performance parameters prior injury and after RTC.

	Prior injury	After RTC	*p* value
Matches played			
2 seasons	27 (IQR: 20–31)	23 (IQR: 13–28)	0.227
1 season	28 (IQR: 24–31)	23 (IQR: 17–31)	0.064
10 match days	9 (IQR: 8–10)	8 (IQR: 6–9)	**0.017**
Minutes played			
2 seasons	1831 (IQR: 1220–2438)	1358 (IQR: 760–2025)	0.139
1 season	2142 (IQR: 1631–2463)	1658 (IQR: 908–2600)	0.082
10 match says	698 (IQR: 459–842)	571 (IQR: 301–702)	**0.004**
Goals scored			
2 seasons	1 (IQR: 0–5)	1 (IQR: 0–4)	0.411
1 season	1 (IQR: 0–5)	1 (IQR: 0–5)	0.326
10 match says	0 (IQR: 0–2)	0 (IQR 0–3)	0.338
Assists made			
2 seasons	2 (IQR: 0–5)	1 (IQR: 0–3)	**0.041**
1 season	2 (IQR 0–5)	2 (IQR: 0–3)	0.250
10 match days	0 (IQR: 0–1)	0 (IQR: 0–1)	0.658

*Note*: All values are given as medians with the respective IQR, statistically significant *p*‐Values are given in bold.

Abbreviations: IQR, interquartile range; RTC, return to competition.

### Qualitative parameters

The Goalimpact increased statistically significantly during the first year after RTC compared with the year prior to injury (141 [IQR: 122–160] vs. 131 [IQR: 115–150], *p* = 0.035) and during the second year following RTC compared with 2 years before (139 [IQR: 124–161] vs. 127 [IQR: 107–153, *p* = 0.003). Similarly, the peak Goalimpact increased significantly during the first year after RTC compared with the year leading up to the injury (145 [IQR: 126–161] vs. 137 [IQR: 120–153], *p* < 0.001) and 2 years after RTC compared with 2 years before (142 [IQR 128–162] vs. 128 [IQR: 118–156], *p *< 0.001) (Table 3).

**Table 3:** Qualitative performance parameters prior injury and after RTC.

	Prior injury	After RTC	*p* value
Goalimpact			
2 years	127 (IQR: 107–153)	139 (IQR: 124–161)	**>0.001**
1 year	131 (IQR: 115–150)	141 (IQR: 122–160)	**0.001**
2 months	138 (IQR: 121–151)	139 (IQR: 121–154)	0.057
1 month	139 (IQR: 121–152)	140 (IQR: 121–152)	0.504
Peak goalimpact			
2 years	128 (IQR: 118–156)	142 (IQR: 128–162)	**<0.001**
1 year	137 (IQR: 120–153)	145 (IQR: 126–161)	**<0.001**
2 months	142 (IQR: 125–155)	143 (IQR: 127–157)	0.147
1 month	143 (IQR: 125–157)	142 (IQR: 127–156)	0.614

*Note*: All values are given as medians with the respective IQR, statistically significant *p*‐Values are given in bold.

Abbreviations: IQR, interquartile range; RTC, return to competition.

### Player‐, match‐ and injury‐related information

The players' median age was 25 years (IQR: 23–26). The most common playing position was centre‐back (*n* = 10, 33%), followed by striker (*n* = 6, 20%). Of the 30 players, 13 (43%) were from the first Bundesliga and 17 (57%) from the second Bundesliga.

Injuries occurred after a median match time of 47 min (IQR: 24–70), with a median time played by the affected player of 42 min (IQR: 16–61). 13 (43%) players suffered their injury during the first half of the season, 17 (57%) players during the second half.

Regarding the mechanism of injury, there were a total of 11 (38%) type 1 shoulder dislocations and 18 (62%) type 2 shoulder dislocations. Conservative treatment was performed on 18 (60%) players, while 12 (40%) underwent surgical treatment. The median layoff time was overall 65 days (IQR: 23–155), with nonoperatively treated players experiencing a median layoff time of 22 days (IQR: 10–34) compared to 112 days (IQR: 92–133) for surgically treated players.

Most player‐, match‐ and injury‐related parameters did not have statistically significant impact on players' performance following RTC (Tables 4a, 4b, 5a, 5b).

**Table 4a:** Quantitative performance parameters matches played and minutes played for player‐, match‐ and injury‐related factors prior injury and after RTC.

	Matches played			Minutes played		
	2 seasons (prior injury vs. after RTC)	1 season (prior injury vs. after RTC)	10 match days (prior injury vs. after RTC)	2 seasons (prior injury vs. after RTC)	1 season(prior injury vs. after RTC)	10 match days (prior injury vs. after RTC)
Player‐related variables						
Age						
<25	6 (IQR: –6 to 20)	–4 (IQR: –5 to 14)	1 (IQR: 0 to 2)	615 (IQR: –498 to 2016)	–289 (IQR: –867 to 902)	238 (IQR: 90 to 303)
>25	1 (IQR: –7 to 8)	5 (IQR: –5 to 11)	1 (IQR: –1 to 1)	–188 (IQR: –713 to 1086)	170 (IQR: –502 to 1167)	96 (IQR: –83 to 170)
*p*‐value	0.286	0.723	1.000	0.191	0.950	0.079
Position played						
Defense	4 (IQR: –7 to 9)	–44 (IQR –7 to 24)	1 (IQR: –2 to 4)	165 (IQR: –774 to 9)	–340 (IQR: –637 to 18)	113 (IQR: –68 to 8)
Midfield	7 (IQR: –2 to 24)	11 (IQR: 4 to 18)	1 (IQR: 0 to 3)	1120 (IQR: –948 to 2159)	963 (IQR: 193 to1306)	189 (IQR: –6 to 243)
Offense	–6 (IQR: –10 to 4)	–3 (IQR: –6 to 8)	1 (IQR: 0 to 1)	–202 (IQR: –663 to 981)	–81 (IQR: –827 to 1528)	179 (IQR: –80 to 319)
*p*‐value	0.250	**0.035**	0.725	0.508	0.185	0.385
League played						
1. Bundesliga	6 (IQR: –7.5 to 15)	2 (IQR: –6.5 to 12)	1 (IQR: –2 to 3)	702 (IQR: –188 to 1737)	263 (IQR: –345 to 1269)	179 (IQR: –100 to 373)
2. Bundesliga	0 (IQR: –7 to 7)	0 (IQR: –4 to 10)	1 (IQR: 0 to 1)	–537 (IQR: –1100 to 1106)	–144 (IQR: –690 to 1026)	139 (IQR: –9 to 203)
*p*‐value	0.650	0.432	0.563	0.142	0.711	0.592
Match‐related variables						
Season						
First half of the season	1 (IQR: –7 to 7)	2 (IQR: –4 to 11)	1 (IQR: 1 to 2)	281 (IQR: –326 to 702)	46 (IQR: 400 to 1528)	193 (IQR: 90 to 246)
Second half of the season	4 (IQR: –7 to 14)	–1 (IQR: –6 to 12)	1 (IQR: 0 to 1)	500 (IQR: –917 to 1646)	91 (IQR: –637 to 1007)	113 (IQR: 102 to 183)
*p*‐value	0.746	0.711	0.145	0.746	0.563	0.103
Injury‐related variables						
Mechanism of injury						
Type 1	4 (IQR: –101 to 5)	2 (IQR: –6 to 13)	1 (IQR: 1 to 1)	702 (IQR: –1002 to 1811)	263 (IQR: –289 to 1375)	165 (IQR: 90 to 234)
Type 2	2 (IQR: –6 to 7)	–3 (IQR: –5 to 9)	1 (IQR: 0 to 2)	54 (IQR: –625 to1085)	–117 (IQR: –735 to 960)	109 (IQR: –88 to 244)
*p*‐value	0.296	0.450	0.488	0.287	0.371	0.631
Type of treatment						
Nonoperative	1 (IQR: –7 to 15)	2 (IQR: –4 to 11)	1 (IQR: 0 to 1)	261 (IQR: –812 to 1774)	128 (IQR: –535 to 955)	139 (IQR: –60 to 244)
Operative	3 (IQR: –1 to 6)	–4 (IQR: –7 to 13)	1 (IQR: 0 to 2)	281 (IQR: –410 to 1091)	46 (IQR: –680 to 1375)	150 (IQR: –36 to 246)
*p* value	0.837	0.692	0.950	0.945	1.000	1.000

*Note*: All values are given as differences of the medians with the respective IQR, statistically significant *p* values are given in bold.

Abbreviations: IQR, interquartile range; RTC, return to competition.

**Table 4b:** Quantitative performance parameters goals made and assists made for player‐, match‐ and injury‐related factors prior injury and after RTC.

	Goals made			Assists made		
	2 seasons (prior injury vs. after RTC)	1 season (prior injury vs. after RTC)	10 match days (prior injury vs. after RTC)	2 seasons (prior injury vs. after RTC)	1 season (prior injury vs. after RTC)	10 match days (prior injury vs. after RTC)
Player‐related variables						
Age						
<25	1 (IQR: –2 to 6)	0 (IQR: –5 to 1)	1 (IQR: 1 to 3)	2 (IQR: –1 to 6)	0 (IQR: –2 to 4)	0 (IQR: –1 to 2)
>25	0 (IQR: –1 to 2)	0 (IQR: 0 to 3)	0 (IQR: –1 to 0)	1 (IQR: –1 to 2)	0 (IQR –1 to 3)	0 (IQR: –1 to 1)
*p*‐value	0.436	0.368	**0.019**	0.265	0.692	0.305
Position played						
Defense	0 (IQR: –1 to 2)	0 (IQR: –1 to 3)	0 (IQR: 0 to 1)	1 (IQR: 0 to 974)	0 (IQR: –1 to 2159)	0 (IQR 0 to 981)
Midfield	1 (IQR: –2 to 2	1 (IQR: 0 to 4)	1 (IQR: –1 to 1)	1 (IQR: –2 to 4)	2 (IQR: –5 to 4)	0 (IQR: –1 to 2)
Offense	2 (IQR: –3 to 8)	1 (IQR: –4 to 5)	3 (IQR: –2 to 4)	2 (IQR: 0 to 3)	3 (IQR: –1 to 4)	–1 (IQR ‐2 to 1)
*p*‐value	0.700	0.133	0.448	0.690	0.404	0.392
League played						
1. Bundesliga	2 (IQR: –1 to 4)	0 (IQR: –1 to 2)	0 (IQR: –1 to 3)	2 (IQR: 0 to 3)	1 (IQR: 0 to 3)	0 (IQR: –1 to 1)
2. Bundesliga	0 (IQR: –2 to 1)	1 (IQR: –2 to 2)	0 (IQR: –1 to 1)	1 (IQR: –1 to 2)	–1 (IQR: –3 to 3)	0 (IQR: –1 to 0)
*p*‐value	0.065	0.902	0.536	0.387	0.198	0.385
Match‐related variables						
Season						
First half of the season	0 (IQR: –1 to 2)	0 (IQR: –1 to 2)	0 (IQR: –1 to 1)	0 (IQR: –1 to 2)	0 (IQR: –2 to 3)	0 (IQR: 0 to 1)
Second half of the season	0 (IQR: –2 to 2)	0 (IQR: –1 to 2)	0 (IQR: –1 to 2)	1 (IQR: –1 to 3)	1 (IQR: –1 to 3)	0 (IQR: –1 to 1)
*p*‐Value	0.781	0.773	0.967	0.781	0.773	0.592
Injury‐related variables						
Mechanism of injury						
Type 1	2 (IQR: 0 to 4)	1 (IQR: 0 to 5)	0 (IQR: –1 to 1)	2 (IQR: 0 to 3)	3 (IQR: –1 to 4)	0 (IQR: –1 to 1)
Type 2	–1 (IQR: –2 to 1)	0 (IQR: –3 to 1)	0 (IQR: –1 to 1)	1 (IQR: –1 to 2)	0 (IQR: –2 to 2)	0 (IQR: –1 to 1)
*p*‐Value	0.092	0.331	0.905	0.694	0.312	0.788
Type of treatment						
Nonoperative	0 (IQR: –1 to 2)	1 (IQR: –1 to 2)	0 (IQR: 0 to 1)	2 (IQR: 0 to 4)	1 (IQR: –1 to 3)	0 (IQR: 0 to 1)
Operative	0 (IQR: –2 to 1)	0 (IQR: –4 to 3)	0 (IQR: –1 to 1)	0 (IQR: –2 to 2)	–1 (IQR: –2 to 3)	0 (IQR: –1 to 1)
*p* value	0.450	0.518	0.518	0.121	0.232	0.491

*Note*: All values are given as differences of the medians with the respective IQR, statistically significant *p* values are given in bold.

Abbreviations: IQR, interquartile range; RTC, return to competition.

**Table 5a:** Qualitative performance parameter ‘Goalimpact’ for player‐, match‐ and injury‐related factors prior injury and after RTC.

	Goalimpact			
	2 years (prior injury vs. after RTC)	1 year (prior injury vs. after RTC)	2 months (prior injury vs. after RTC)	1 month (prior injury vs. after RTC)
Player‐related variables				
Age				
<25	–2 (IQR: –10 to 6)	–5 (IQR: –9 to 3)	0 (IQR: –3 to 2)	–1 (IQR: –3 to 2)
>25	–20 (IQR: –32 to –6)	–12 (IQR: –16 to –7)	–2 (IQR: –5 to 2)	–1 (IQR: –3 to 2)
*p*‐Value	**0.003**	**0.003**	0.602	1.000
Position played				
Defense	–12 (IQR: –28 to –4)	–10 (IQR: –15 to –5)	–1 (IQR: –5 to 1)	1 (IQR: –1 to 2)
Midfield	–10 (IQR: –23 to 9)	–10 (IQR: –14 to –5)	–1 (IQR: –5 to 2)	–1 (IQR: –4 to 1)
Offense	–11 (IQR: –33 to –3)	–9 (IQR: –14 to –1)	–1 (IQR: –4 to 2)	–1 (IQR: –4 to 3)
*p*‐Value	0.693	0.950	0.723	0.476
League played				
1. Bundesliga	–11 (IQR: –21 to –1)	–9 (IQR: –13 to –4)	1 (IQR: –3 to 1)	–1 (IQR: –2 to 1)
2. Bundesliga	–15 (IQR: –30 to –3)	–11 (IQR: –15 to –5)	–2 (IQR: –5 to 1)	0 (IQR: –4 to 2)
*p*‐Value	0.589	1.000	0.245	0.536
Match‐related variables				
Season				
First half of the season	–14 (IQR: –23 to –3)	–10 (IQR: –14 to 1)	–2 (IQR: –5 to 1)	–1 (IQR: –3 to 1)
Second half of the season	–11 (IQR: –28 to –2)	–9 (IQR: –14 to –5)	0 (IQR: –4 to 2)	0 (IQR: –3 to 3)
*p*‐Value	0.650	0.680	0.509	0.483
Injury‐related Variables				
Mechanism of injury				
Type 1	–18 (IQR: –31 to –11)	–10 (IQR: –11 to –4)	1 (IQR: –4 to 1)	–1 (IQR: –2 to 1)
Type 2	–6 (IQR: –23 to –2)	–11 (IQR: –4 to 1)	‐–3 (IQR: –5 to 2)	–1 (IQR: –4 to 2)
*p*‐Value	0.122	0.877	0.317	0.674
Type of treatment				
Nonoperative	–12 (IQR: –23 to –4)	–10 (IQR: –14 to –6)	–2 (IQR: –5 to 1)	–1 (IQR: –3 to 2)
Operative	–7 (IQR: –30 to 13)	–8 (IQR: –15 to 1)	0 (IQR: –4 to 2)	0 (IQR: –4 to 2)
*p*‐Value	0.774	0.632	0.305	0.325

*Note*: All values are given as differences of the medians with the respective IQR, statistically significant *p* values are given in bold.

Abbreviations: IQR, interquartile range; RTC, return to competition.

**Table 5b:** Qualitative performance parameter ‘peak Goalimpact’ for player‐, match‐ and injury‐related factors prior injury and after RTC.

	Goalimpact peak			
	2 years (prior injury vs. after RTC)	1 year (prior injury vs. after RTC)	2 months (prior injury vs. after RTC)	1 month (prior injury vs. after RTC)
Player‐related variables				
Age				
<25	–4 (IQR: –8 to 0)	–2 (IQR: –7 to 1)	–1 (IQR: –4 to 1)	–1 (IQR: –3 to 1)
>25	–15 (IQR: –27 to –2)	–10 (IQR: –14 to –3)	–1 (IQR: –4 to 2)	0 (IQR: –3 to 2)
*p*‐Value	0.076	**0.035**	0.692	0.787
Position played				
Defense	–4 (IQR: –17 to 0)	–6 (IQR: –13 to –1)	–1 (IQR: –4 to 0)	1 (IQR: –2 to 2)
Midfield	–7 (IQR: –21 to 1)	–10 (IQR: –12 to –2)	–4 (IQR: –5 to 1)	–1 (IQR: –3 to 1)
Offense	–9 (IQR: –29 to –2)	–7 (IQR: –13 to 1)	0 (IQR: –2 to 5)	–1 (IQR: –4 to 3)
*p*‐Value	0.701	0.774	0.376	0.417
League played				
1. Bundesliga	–4 (IQR: –16 to 2)	–7 (IQR: –11 to –1)	0 (IQR: –3 to 3)	0 (IQR: –1 to 3)
2. Bundesliga	–8 (IQR: –22 to –2)	–8 (IQR: –13 to –1)	–3 (IQR: –4 to 0)	–1 (IQR: –4 to 1)
*p*‐Value	0.249	0.869	0.183	0.592
Match‐related variables				
Season				
First half of the season	–4 (IQR: –20 to 0)	–8 (IQR: –13 to 1)	–2 (IQR: –4 to 0)	–1 (IQR: –3 to 1)
Second half of the season	–8 (IQR: –18 to 0)	–7 (IQR: –13 to –2)	–1 (IQR: –4 to 3)	0 (IQR: –3 to 3)
*p*‐Value	0.846	0.902	0.341	0.363
Injury‐related variables				
Mechanism of injury				
Type 1	–15 (IQR: –18 to –3)	–6 (IQR: –10 to 0)	0 (IQR: –1 to 2)	0 (IQR: –2 to 2)
Type 2	–4 (IQR: –20 to 0)	–9 (IQR: –14 to –2)	–3 (IQR: –4 to 0)	–1 (IQR: –4 to 2)
*p*‐Value	0.276	0.412	0.122	0.465
Type of treatment				
Nonoperative	–6 (IQR: –17 to 0)	–7 (IQR: –13 to –1)	–1 (IQR: –4 to 0)	–1 (IQR: –3 to 2)
Operative	–8 (IQR: –26 to 2)	–8 (IQR: –14 to –2)	–2 (IQR: –3 to 2)	0 (IQR: –4 to 1)
*p*‐Value	0.877	0.950	0.465	0.368

*Note*: All values are given as differences of the medians with the respective IQR, statistically significant *p*‐Values are given in bold. Abbreviations: IQR, interquartile range; RTC, return to competition.

## DISCUSSION

The main findings of this study are: (1) Players participate in fewer matches and accumulate fewer minutes in the 10 matches following RTC compared to the 10 matches prior to injury. (2) The qualitative performance parameters Goalimpact and Peak Goalimpact initially remain similar and tend to increase the first and second year following RTC. (3) Player‐, match‐ and injury‐related parameters generally show no statistically significant impact on performance following an in‐match shoulder dislocation in professional football. These findings suggest that professional football players maintain their performance level after first‐time shoulder dislocations, with respect to the period studied.

Players experienced a notable decrease in match participation during the first 10 matches following RTC. Potential reasons for this decline are manifold and may include regular team dynamics, individual lack of match play, but also psychological factors such as fear of re‐dislocation. Psychological factors are often underestimated in the context of RTC [3, 15]. A recent systematic review by Gibbs et al. has identified fear of re‐injury as the most frequently reported barrier to RTC among patients who have experienced a shoulder instability event [8].

Another key factor contributing to decreased match participation is the shift in team dynamics during an injured player's absence [5]. Tactical adjustments made by coaches in their absence may temporarily disrupt the player's performance upon return, requiring time for adaptation. Additionally, changes in coaching or team staff during the layoff time can pose further uncertainness, making the reintegration process even more challenging for returning players [2, 6, 16]. Furthermore, the load control following an injury often requires a gradual return to full training and match play is likely necessary to prevent re‐injury and may explain the reduced number of matches and minutes played during the initial phase of RTC. Hägglund et al. demonstrated that a controlled, graduated rehabilitation program reduces re‐injury risk by 66% for all injury locations and 75% for lower limb injuries in professional football [11].

While quantitative parameters showed a statistically significant decline immediately after RTC, qualitative parameters initially remained comparable and even showed improvement over time. This observation suggests that shoulder dislocations may not impede a player's long‐term development. This is in line with findings from a recent study that found that all 19 goalkeepers from 5 European top leagues who underwent surgical treatment following a shoulder dislocation returned to play without a relevant change in the individual player's market value [18] However, the Goalimpact has not been previously investigated in professional football, further research—preferably a prospective analysis of player development over time—is needed to confirm these findings. Future studies should compare players with shoulder instability to matched players without such instability to confirm the validity of the Goalimpact scoring metric and its potential implications for predicting future player performance.

Regarding injury‐related factors, neither the mechanism of injury (direct contact vs. catching a fall) nor the subsequent treatment (nonoperative vs. operative) showed an impact on either quantitative or qualitative performance parameters. Mechanisms of injury have shown to influence RTC in professional football. A study on over 2000 thigh muscle injuries over a 10‐year period found that direct‐contact thigh muscle injuries result in shorter recovery times and a quicker return to full activity compared to indirect injuries [23]. Considering the limited number of injuries included as well as the novelty of the proposed classification of the different mechanisms of injury, future studies should focus on replicating these findings in larger patient cohorts.

The ideal treatment following first‐time in‐match shoulder dislocations in professional football remains a topic of debate [9, 21]. Regarding posttraumatic performance, the findings suggest that both treatment approaches—nonoperative and surgical—may be equally effective in facilitating RTC and maintaining player performance. This insight is particularly important for clinical practice, as it underlines the importance of individualised rehabilitation protocols rather than solely focusing on physical intervention.

Conversely, different outcomes have been reported in sports with greater upper‐extremity involvement: Swindell et al. found that surgical treatment for shoulder dislocations in the NHL resulted in fewer recurrent injuries than conservative treatment and recurrent shoulder dislocations lead to significantly more missed career games, indicating that hockey players could benefit from surgical intervention [22].

In a study on 60 NBA players over a 30‐year period, Li et al. observed a direct impact of surgical intervention for shoulder instability on players' performance metrics—such as maintaining their player efficiency rating (PER), games played and win share performance characteristics—while nonoperatively treated players experienced declines in these parameters [14]. According to Li et al., players who underwent surgery for shoulder dislocations participated in significantly more games in the three seasons post‐injury compared to those who did not receive surgical intervention. Concerning PER and offensive win shares, a significant decrease among players who were treated conservatively in the season following the injury was noted [14]. These findings illustrate the sport‐specific impact of upper‐extremity injuries. In sports, such as ice hockey and basketball, where upper‐extremity function is crucial for performance, surgical stabilisation has been associated with superior return to play outcomes. In contrast, the present study did not demonstrate a clear performance advantage for operative over nonoperative management in professional football. Furthermore, the shorter layoff observed among conservatively treated players represents an important consideration in clinical decision‐making. Collectively, these observations emphasise the necessity for individualised, sport‐specific treatment decisions based on injury characteristics, player demands and recovery goals.

### Limitations

Several limitations must be acknowledged in this study. Although the incidence of shoulder dislocations in professional football is increasing, overall, this injury has a relatively low incidence, with an occurrence rate of 9.1 per 1000 matches played [4, 21]. As such, to ensure comparability with similar studies on different injuries, the cohort of the present study was extended by including both top German football leagues over a study period of 10 years, resulting in a total cohort of 30 players [7, 14, 19]. Since all available players suffering a shoulder dislocation within the study period were included, a priori sample size calculation based on power analysis was not feasible; thus, the absence of statistical power calculation represents a limitation of the study.

Shoulder dislocations carry the risk of concomitant injuries to soft tissues and bone, such as Hill‐Sachs lesions, and surgical treatment options can range from soft‐tissue stabilisation to bone block stabilisation [9]. As medical imaging and operative data are confidential and thus not publicly available, these factors could not be considered when analysing parameters affecting posttraumatic performance. In addition, publication bias cannot be fully excluded, as media sources may preferentially report more severe or high‐profile cases. Nevertheless, it has been established that for professional football players in Germany, medical studies based on public data are sufficiently reliable [13]. Additionally, as a systematic video analysis has shown, these shoulder dislocations almost exclusively follow a set mechanism of injury, suggesting potentially similar concomitant injury patterns [20]. Recurrent shoulder instability may represent another important factor influencing long‐term performance and future prospective studies should focus on its impact.

It cannot be ruled out that other factors, such as infections or additional injuries, may have influenced the layoff time. Some players sustained multiple musculoskeletal injuries during the study period.

Finally, the retrospective nature of this study presents several limitations. Since retrospective studies rely on existing data, not all relevant factors that may influence performance outcomes can be captured. It was assumed that all shoulder dislocations and their type of treatment in the first and second Bundesliga were accurately reported [13]. To minimise the influence of potential confounding variables on the results, performance metrics dependent on age, position, league affiliation, point in the season, mechanism of injury and type of treatment were included. Nonetheless, this approach does not account for all potential confounding variables. Therefore, while our findings offer important insights into how shoulder dislocations affect player performance, they should be interpreted with caution and validated in future prospective studies.

## CONCLUSION

Following an in‐match shoulder dislocation in professional football, players participate in fewer matches and less minutes during the first 10 matches following RTC compared with the 10 matches pre‐injury. However, in the mid‐to‐long term, quantitative performance parameters showed no significant decline, suggesting that players are generally able to maintain their performance levels over time. Qualitative performance parameters remained largely unaffected and, overall, player‐, match‐ and injury‐related factors did not significantly influence post‐injury performance. Future studies should address the limitations of this retrospective analysis through prospective research that includes imaging analysis, patient‐reported outcomes and extended follow‐up periods.

## AUTHOR CONTRIBUTIONS

Blanca Julie Degener and Kristian Nikolaus Schneider designed the study. Blanca Julie Degener, Christoph Theil, Georg Gosheger, Patrick May, Jörg Seidel Tim Schachtrup, Theodoros Zafeiris and Kristian Nikolaus Schneider collected and analysed the data. Blanca Julie Degener, Christoph Theil, Georg Gosheger, Patrick May, Jörg Seidel and Kristian Nikolaus Schneider were responsible for data management and preparation of figures. Blanca Julie Degener and Kristian Nikolaus Schneider wrote the manuscript. Christoph Theil and Georg Gosheger helped with data analysis and with editing of the manuscript. All authors read and approved the final manuscript.

## CONFLICT OF INTEREST STATEMENT

The authors declare no conflicts of interests.

## ETHICS STATEMENT

The study was approved by the Ethikkommission der Ärztekammer Westfalen/Lippe (reference number 2023‐687‐f‐S) and performed in accordance with the Declaration of Helsinki. Patient consent was waived due to ethics committee approval. All figures and tables are exclusively original, there is no material from other sources. The authors affirm that the study's supporting data are contained within the article.

## Data Availability

The data that support the findings of this study are contained within the manuscript. The raw data can be obtained from the corresponding author on reasonable request.

## References

[1] Aus der Fünten K , Tröß T , Hadji A , Beaudouin F , Steendahl IB , Meyer T . Epidemiology of football injuries of the German Bundesliga: a media‐based, prospective analysis over 7 consecutive seasons. Sports Med Open. 2023;9:20. 36867257 10.1186/s40798-023-00563-x PMC9982794

[2] Dönmez G , Kudaş S , Yörübulut M , Yıldırım M , Babayeva N , Torgutalp ŞŞ . Evaluation of muscle injuries in professional football players: does coach replacement affect the injury rate? Clin J Sport Med. 2020;30:478–483. 30113968 10.1097/JSM.0000000000000640

[3] Dunlop G , Ivarsson A , Andersen TE , Brown S , O'Driscoll G , Lewin C , et al. Examination of the validity of the Injury‐Psychological Readiness to Return to Sport (I‐PRRS) scale in male professional football players: a worldwide study of 29 professional teams. J Sports Sci. 2023;41:1906–1914. 38269550 10.1080/02640414.2024.2307764

[4] Ekstrand J , Hägglund M , Waldén M . Injury incidence and injury patterns in professional football: the UEFA injury study. Br J Sports Med. 2011;45:553–558. 19553225 10.1136/bjsm.2009.060582

[5] Ekstrand J , Hägglund M , Waldén M , Gauffin H , Baudot C , Biosca P , et al. Higher level of communication between the medical staff and the performance staff is associated with a lower hamstring injury burden: a substudy on 14 teams from the UEFA Elite Club Injury Study. BMJ Open Sport Exerc Med. 2025;11:e002182. 10.1136/bmjsem-2024-002182 PMC11877254 40041682

[6] Ekstrand J , van Zoest W , Gauffin H . Changes in head staff members in male elite‐level football teams are associated with increased hamstring injury burden for that season: the UEFA Elite Club Injury Study. BMJ Open Sport Exerc Med. 2023;9:e001640. 10.1136/bmjsem-2023-001640 PMC10660205 38022762

[7] Farinelli L , Csapo R , Meena A , Abermann E , Hoser C , Fink C . Concomitant iInjuries associated with ACL rupture in elite professional alpine Ski racers and soccer players: a comparative study with propensity score matching analysis. Orthop J Sports Med. 2023;11:23259671231192127. 10.1177/23259671231192127 37655251 PMC10467387

[8] Gibbs D , Mallory N , Hoge C , Jones G , Bishop J , Cvetanovich G , et al. Psychological factors that affect return to sport after surgical intervention for shoulder instability: a systematic review. Orthop J Sports Med. 2023;11:23259671231207649. 10.1177/23259671231207649 38035214 PMC10686029

[9] Goth AP , Klug A , Gosheger G , Hiort ML , Akgün D , Schneider KN . Traumatic anterior shoulder dislocation: epidemiology, diagnosis, and treatment. Dtsch Arztebl Int. 2025;122(4):89–95. 39836468 10.3238/arztebl.m2024.0254 PMC12439206

[10] Grassi A , Macchiarola L , Filippini M , Lucidi GA , Della Villa F , Zaffagnini S . Epidemiology of anterior cruciate ligament injury in Italian first division soccer players. Sports Health Multidiscip Approach. 2020;12:279–288. 10.1177/1941738119885642 PMC7222666 31800358

[11] Hägglund M , Walden M , Ekstrand J . Lower reinjury rate with a coach‐controlled rehabilitation program in amateur male soccer: a randomized controlled trial. Am J Sports Med. 2007;35:1433–1442. 17369558 10.1177/0363546507300063

[12] Hägglund M , Waldén M , Magnusson H , Kristenson K , Bengtsson H , Ekstrand J . Injuries affect team performance negatively in professional football: an 11‐year follow‐up of the UEFA champions league injury study. Br J Sports Med. 2013;47:738–742. 23645832 10.1136/bjsports-2013-092215

[13] Krutsch V , Grechenig S , Loose O , Achenbach L , Zellner J , Striegel H , et al. Injury analysis in professional soccer by means of media reports—only severe injury types show high validity. Open Access J Sports Med. 2020;11:123–131. 32884370 10.2147/OAJSM.S251081 PMC7431944

[14] Li NY , Lemme NJ , Defroda SF , Nunez E , Hartnett DA , Owens BD . Performance after operative versus nonoperative management of shoulder instability in the National Basketball Association. Orthop J Sports Med. 2019;7:2325967119889331. 10.1177/2325967119889331 31858014 PMC6913052

[15] Pasqualini I , Rossi LA , Hurley ET , Turan O , Tanoira I , Ranalletta M . Shoulder instability‐return to sports after injury scale shows that lack of psychological readiness predicts outcomes and recurrence following surgical stabilization. Arthroscopy J Arthroscopic Relat Surg. 2024;40:2815–2824. 10.1016/j.arthro.2024.04.030 38735414

[16] Petrov D , Parpa K , Michaelides M . The impact of managerial changes on physical performance in elite soccer players. Sports. 2025;13(7):213. 40711098 10.3390/sports13070213 PMC12298339

[17] Pfirrmann D , Herbst M , Ingelfinger P , Simon P , Tug S . Analysis of injury incidences in male professional adult and elite youth soccer players: a systematic review. J Athl Train. 2016;51:410–424. 27244125 10.4085/1062-6050-51.6.03 PMC5013706

[18] Redler A , Annibaldi A , Benelli C , Carrozzo A , Carta B , Iorio R , et al. The impact of anterior shoulder dislocation in elite soccer goalkeepers. Orthop J Sports Med. 2025;13:23259671251358403. 10.1177/23259671251358403 40771888 PMC12326124

[19] Rekik R , Bahr R , Cruz F , Read P , Whiteley R , D'hooghe P , et al. Mechanisms of ACL injuries in men's football: a systematic video analysis over six seasons in the Qatari professional league. Biol Sport. 2023;40:575–586. 37077782 10.5114/biolsport.2023.118024 PMC10108748

[20] Schneider KN , Schachtrup T , Gosheger G , Hiort ML , Degener BJ , Zafeiris T , et al. Systematic video analysis of shoulder dislocations in professional male football (soccer): injury mechanisms, situational and kinematic patterns. J Exp Orthop. 2024;11:e70121. 10.1002/jeo2.70121 39703833 PMC11656221

[21] Schneider KN , Zafeiris T , Gosheger G , Klingebiel S , Rickert C , Schachtrup T , et al. Shoulder dislocations in professional male football (soccer): a retrospective epidemiological analysis of the German Bundesliga from season 2012/2013 until 2022/2023. Knee Surg Sports Traumatol Arthrosc. 2024;32:1591–1598. 38643395 10.1002/ksa.12199

[22] Swindell HW , McCormick KL , Tedesco LJ , Herndon CL , Ahmad CS , Levine WN , et al. Shoulder instability, performance, and return to play in National Hockey League players. JSES Int. 2020;4:786–791. 33345216 10.1016/j.jseint.2020.08.008 PMC7738589

[23] Ueblacker P , Müller‐Wohlfahrt H‐W , Ekstrand J . Epidemiological and clinical outcome comparison of indirect (‘strain’) versus direct (‘contusion’) anterior and posterior thigh muscle injuries in male elite football players: UEFA elite league study of 2287 thigh injuries (2001–2013). Br J Sports Med. 2015;49:1461–1465. 25755277 10.1136/bjsports-2014-094285

